# The Leuven Embedded Figures Test (L-EFT): measuring perception, intelligence or executive function?

**DOI:** 10.7717/peerj.4524

**Published:** 2018-03-26

**Authors:** Hanne Huygelier, Ruth Van der Hallen, Johan Wagemans, Lee de-Wit, Rebecca Chamberlain

**Affiliations:** 1 Laboratory for Experimental Psychology, KU Leuven, Leuven, Belgium; 2 Cognitive Aspects of Psychopathology, Department of Psychology, Education & Child Studies, Erasmus University Rotterdam, Rotterdam, Netherlands; 3 Cognition and Language Sciences, Chandler House, University College London, London, United Kingdom; 4 Department of Psychology, Goldsmiths College, University of London, London, United Kingdom

**Keywords:** Intelligence, Perceptual style, Executive functions, Weak central coherence, Embedded figures, EFT

## Abstract

Performance on the Embedded Figures Test (EFT) has been interpreted as a reflection of local/global perceptual style, weak central coherence and/or field independence, as well as a measure of intelligence and executive function. The variable ways in which EFT findings have been interpreted demonstrate that the construct validity of this measure is unclear. In order to address this lack of clarity, we investigated to what extent performance on a new Embedded Figures Test (L-EFT) correlated with measures of intelligence, executive functions and estimates of local/global perceptual styles. In addition, we compared L-EFT performance to the original group EFT to directly contrast both tasks. Taken together, our results indicate that performance on the L-EFT does not correlate strongly with estimates of local/global perceptual style, intelligence or executive functions. Additionally, the results show that performance on the L-EFT is similarly associated with memory span and fluid intelligence as the group EFT. These results suggest that the L-EFT does not reflect a general perceptual or cognitive style/ability. These results further emphasize that empirical data on the construct validity of a task do not always align with the face validity of a task.

## Introduction

**Figure 1:**
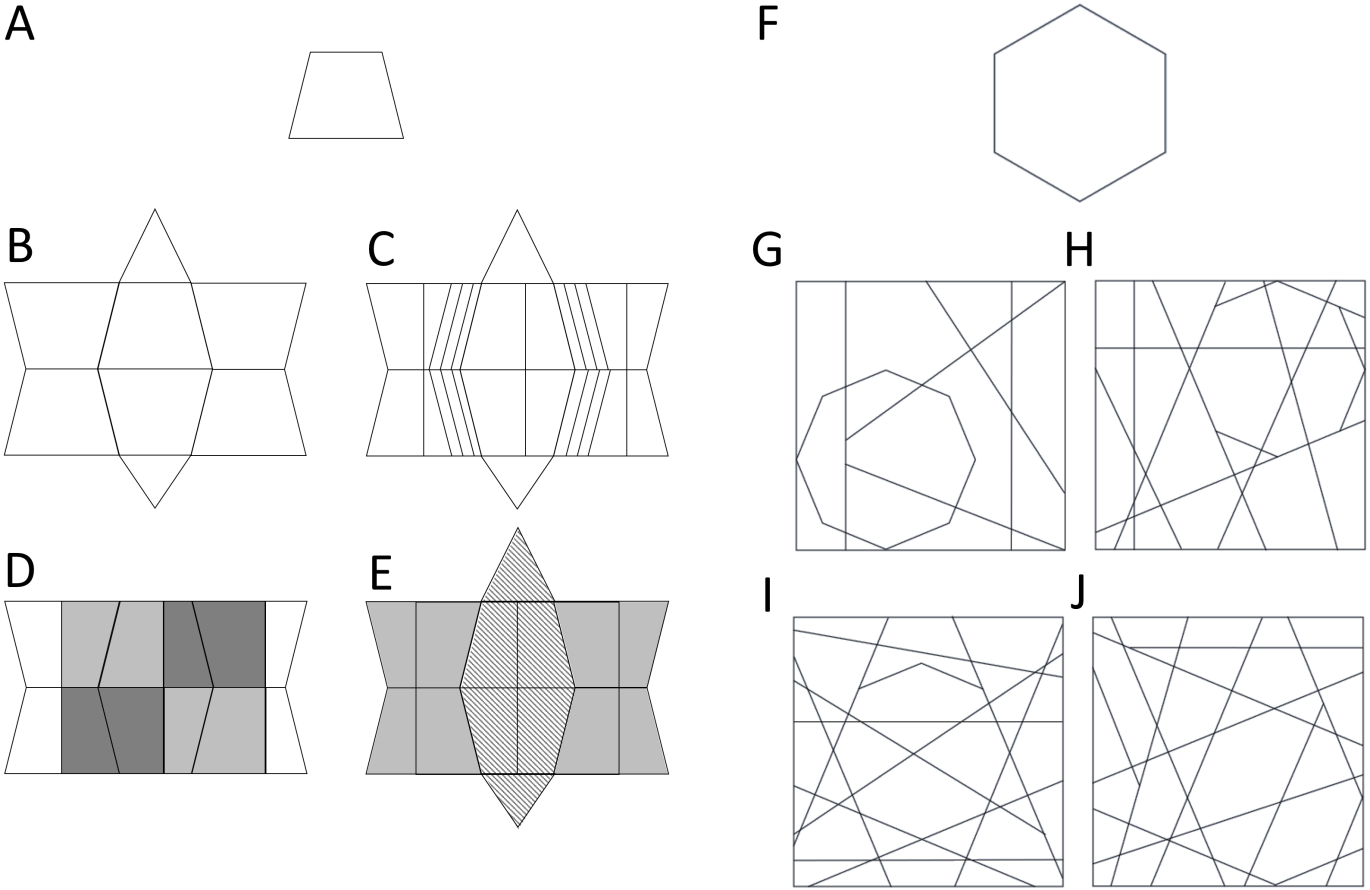
The embedding principles of the Group EFT and L-EFT. Examples of target shapes and context shapes according to the embedding principles used in the G-EFT (A–E) and L-EFT (F–J). The figures A to E are constructed by the authors of this paper to resemble the shapes of the G-EFT developed by Witkin for illustration purposes (the real G-EFT figures are copyright protected). The simple line shape (A) is embedded within four context shapes through different embedding principles: line continuation (B), parallel lines (C), shading to create incongruent segmentation (D) and shading to create incongruent figure-ground interpretations (E). These different embedding principles are used in different trials of the G-EFT in combination with different target shapes (no systematic factorial combination). In (F) an example target stimulus and in (G to J) four embedding contexts containing the target shape are presented. The target is increasingly embedded in the context shape through a higher number of continued lines. Target shape and number of continued lines are combined in a factorial way in the L-EFT.

Most theories, models and experiments in cognitive psychology aim to reveal general principles of mental functioning. However, while general principles are to be acknowledged, important inter-individual differences exist regarding cognitive abilities and styles of information processing (De Wit & Wagemans, 2015). One important contribution to research on individual differences in visual perception and cognition was made by Herman Witkin. In 1950, Witkin developed the Embedded Figures Test (EFT) to measure an information processing style that was either *field-dependent* or *field*-*independent* (Witkin, 1950). In the EFT observers must find a simple line drawing (target shape) within a more complex line drawing (Figs. 1A–1E). The concept of field-(in)dependence referred to individual differences in the (in)sensitivity to information from a broader context. Shortly after, Adevai, Silverman & Gough (1968) revealed that people who were better at detecting the orientation of a rod in a tilted frame were also better at finding target shapes in embedded figures. Witkin assumed that the correlation between these two tests revealed an underlying cognitive style that could impact a wide array of domains, especially with regard to education (Witkin et al., 1975). Witkin and colleagues hypothesized that individuals with a field-independent style were generally more analytic in their approach and would therefore be better suited for curricula and jobs that were focused on mathematics, sciences and engineering than field-dependent individuals.

Later, research on Autism Spectrum Disorder (ASD) revealed that individuals with ASD outperformed typically developing controls on the EFT (Jolliffe & Baron-Cohen, 1997; Ring et al., 1999; Shah & Frith, 1983). This finding shifted the interpretation of good performance on the EFT from reflecting a field-independent *cognitive* style to a reflection of a specific type of *perceptual* processing, which was referred to as weak central coherence (Happé & Frith, 2006) or enhanced local processing (Mottron et al., 2006).

However, empirical work by Milne & Szczerbinski (2009) questions to what extent the EFT measures the aforementioned constructs. Milne and Szczerbinski performed a factor analysis on a wide range of tasks assumed to measure enhanced local processing or weak central coherence (including the group EFT; Witkin et al., 1971) to test whether a common factor underlies these different local-global tasks. Their results suggested that individual differences on the EFT reflected a factor called *disembedding*, but did not reflect a general local or global perceptual style. The disembedding factor also correlated with coherent motion thresholds and intelligence in their student sample.

To further obfuscate the conceptual clarity of the EFT, good performance on the EFT has consistently been linked to general intelligence and, in more recent years, has also been interpreted as a measure of executive functions (Brosnan et al., 2002; Goodenough & Karp, 1961; Richardson & Turner, 2000; Roberge & Flexer, 1981). John Duncan (2013) has even explicitly used the EFT to illustrate the types of problem solving that are typically involved in tests of fluid intelligence. The theoretical account that he has put forward is supported by numerous observations. For example, Bölte et al. (2007) reported a high correlation (*r* = .63) between nonverbal IQ (using the Block Design subscale of the Wechsler Intelligence Scales) and EFT performance in a sample of high-functioning autism, schizophrenics, depressed and healthy individuals and McKenna (1984) listed a considerable number of studies that found moderate to large correlations between measures of IQ and different embedded figures tasks that were robust across various types of samples. Although the EFT has also been interpreted as a measure of executive functions (Brosnan et al., 2002), there have been no studies, to our knowledge, that explicitly measured the association between EFT performance and executive functions.

The current studies were motivated by our limited understanding of what may drive individual differences in EFT performance. To study this, the Leuven Embedded Figures Test (L-EFT), a computerised EFT with a well-controlled stimulus set regarding the perceptual factors involved in embedding was used (De Wit et al., 2017). We evaluated to what extent the L-EFT relates to a local or global perceptual style and to measures of fluid intelligence and executive functions. In addition, the L-EFT is compared to Witkin’s group EFT in order to assess the extent to which these reflect (dis)similar underlying perceptual or cognitive processes. In summary, these studies provide further clarification on the extent to which EFTs measure local/global perceptual style, fluid intelligence and executive functions.

In Study 1, we set out to investigate whether the variance in performance on the L-EFT could be explained by the variance in performance on two other related perceptual tests that have often been assumed to measure a common local or global bias (namely, a variant of the Navon hierarchical letter task and a coherent motion task). Based on a previous study investigating variants of the L-EFT in relation to hierarchical perceptual processing in the Navon task, it was predicted that there would be little correlation between local-global perceptual performance and performance on the L-EFT (Chamberlain et al., 2017). In Study 2, we set out to investigate to what extent individual differences on the L-EFT could be predicted by performance on an array of different executive function (EF) tasks and fluid intelligence. On the basis of previous research (Goodenough & Karp, 1961; McKenna, 1984; Richardson & Turner, 2000; Roberge & Flexer, 1981) it was predicted that there would be moderate correlations between fluid intelligence and performance on the L-EFT. The moderate correlation between the EFT and fluid intelligence would reflect residual problem-solving processes required to perform the tasks. For the EFs we explored whether performance on the L-EFT was associated to memory span, inhibition and cognitive flexibility.

In Study 3, we compared the L-EFT to Witkin’s original group EFT (G-EFT). The two tasks differ in two important aspects. The perceptual factors that make the target shape more difficult to find (*embedding principles*) are systematically manipulated in the L-EFT and their effect on difficulty has been explicitly studied which is not the case for the G-EFT. The main embedding principle in the L-EFT is the number of lines continued from the target into the context shape (De Wit et al., 2017; see Figs. 1F–1J for an example). The embedding principles in the G-EFT, as evaluated on the basis of our visual inspection, include manipulations of 3D shapes that conflict with the 2D target shape, shading to create conflicting figure-ground interpretations (Fig. 1E), shading to create conflicting segmentation (Fig. 1D), line continuation (Fig. 1B), mirror symmetry in the context shape and adding lines that are parallel with the target lines (Fig. 1C).

The L-EFT and G-EFT also differ in the task procedure. In the L-EFT the target shape is presented simultaneously with the embedding context, while in the G-EFT the target shape is not presented simultaneously with the embedding context. Due to this difference participants may need to hold the target shape in memory for longer periods of time in the G-EFT than in the L-EFT. Therefore, we predict that memory span is more strongly associated to the G-EFT than the L-EFT.

In addition, Study 2 and 3 include an evaluation of the split-half and test-retest reliability of the L-EFT to test whether the variance that is picked up by the L-EFT remains consistent within the test and across different test moments.

## Study 1

Study 1 aimed to evaluate to what extent the variance in performance on the L-EFT could be explained by the variance in performance on a variant of the Navon hierarchical letter task and a Coherent Motion task. The Navon hierarchical letter task was selected due to its popularity as a tool to measure local versus global perceptual style in ASD (Happé & Frith, 2006). The Coherent Motion task was selected as it has previously been shown to be associated with a disembedding factor on which the G-EFT loaded significantly (Milne & Szczerbinski, 2009). If L-EFT performance reflects a local/global perceptual style, performance on the L-EFT should be predicted by the performance on the Navon and Coherent Motion task. Furthermore, the Navon task included conditions in which participants were instructed to selectively attend the local or global level of the hierarchical letter. Thus, if the L-EFT reflects a local perceptual style we would predict a stronger association between good L-EFT performance and good performance on the local than the global attention condition of the Navon Selective Attention Task (NSAT). If the L-EFT reflects a global perceptual style we would predict a stronger association between good L-EFT performance and good performance on the global than the local attention condition of the NSAT. Good performance on the Coherent Motion task is typically interpreted as a reflection of a global perceptual style. Therefore, if good L-EFT performance reflects a global perceptual style we predict a positive association between good performance on the Coherent Motion task and good performance on the L-EFT.

### Methods

#### Participants

A group of 62 undergraduate psychology students took part in this study for course credits. The median age was 19 years (*SD* = 3.13). The sample was primarily female (85%). All procedures performed in this study were in accordance with the ethical standards of the institutional ethical committee and approved by the ethical committee of the KU Leuven (approval number: S58409) as well as in accordance with the 1964 Helsinki declaration and its later amendments or comparable ethical standards. Written informed consent for each participant was obtained prior to testing.

#### Instruments

##### Leuven Embedded Figures Test (L-EFT).

**Figure 2:**
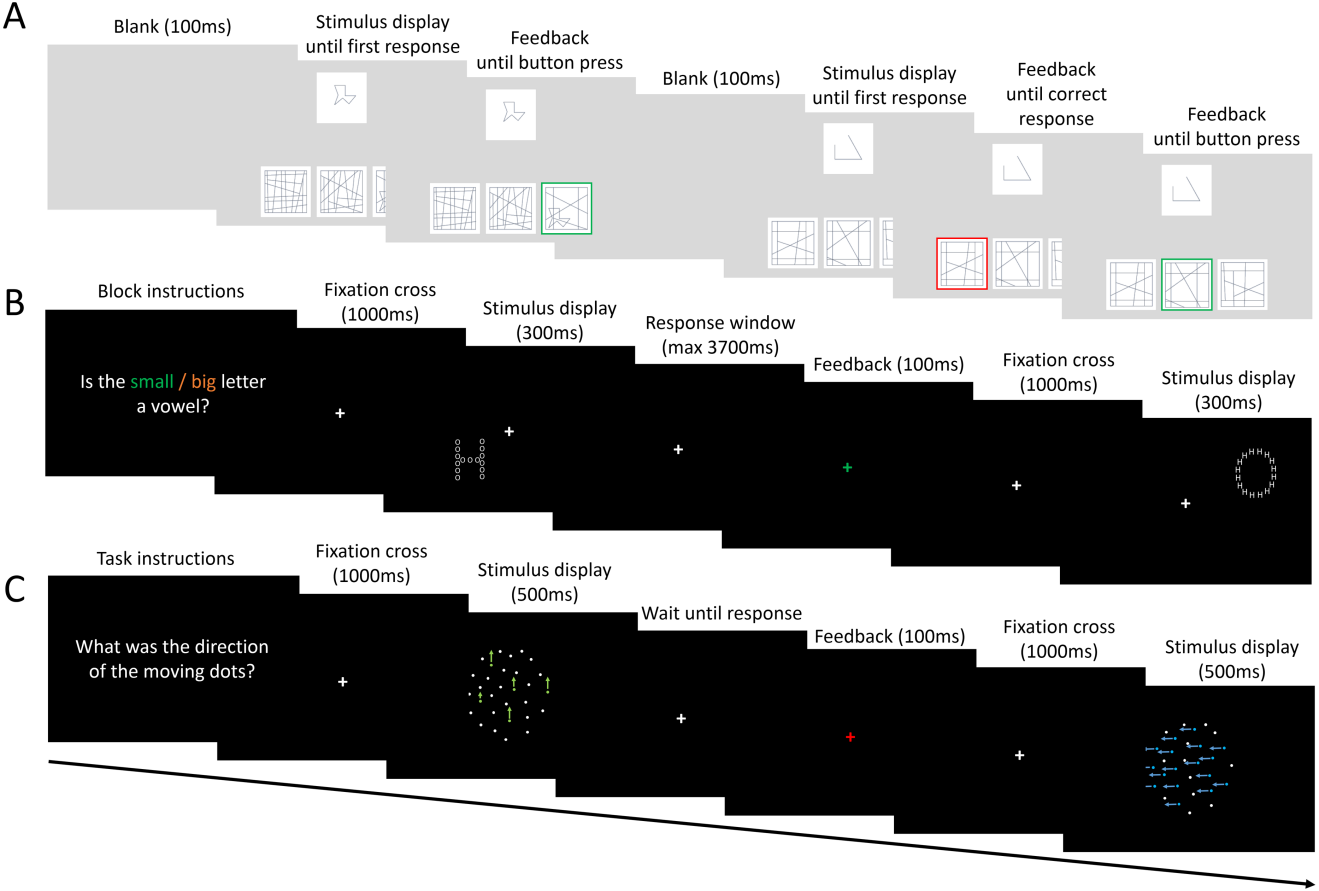
Visualization of the tasks used in Study 1. The task procedure of the L-EFT (A), NSAT (B) and CM task (C) as used in Study 1. The colors in the CM task are only added for illustration purposes. In the experiment all dots had the same color.

The stimulus set of the L-EFT consisted of 16 different target shapes (simple line drawings) each embedded to a varying degree in a context shape, producing 64 unique trials. Participants were asked to perform a matching-to-sample task and were presented with one target shape (on the top of the screen) and three context shapes (on the bottom of the screen) simultaneously (Fig. 2A). The target and context shapes had a size of 3 cm^2^. The target shape was presented in the middle on top of the screen and the context shapes were presented next to each other and 7 cm below the target shape. Of the three context shapes presented, one contained the target while the other two were distractor contexts. The context shape with the target always contained the target shape with the same scale and orientation as the target shape presented on top of the screen. Participants were asked to choose which context shape contained the target as quickly and accurately as possible by clicking on the response alternative with the computer mouse. The stimulus displays were presented until the participant provided a correct response. If they provided a wrong response, feedback was given and they were prompted to give a new response until they chose the correct context shape. All 64 trials were presented in a random order. The entire task takes 5 to 10 min to complete. Stimulus presentation and response registration were controlled using custom software written in C# developed in Visual Studio.

##### Navon Selective Attention Task (NSAT).

A white global letter with a size of 3.5 cm^2^ made up of local letters was presented 2.5 cm from the fixation cross against a black background (Fig. 2B). The global and local letters could be 1 of 5 consonants (C, D, F, H, T) or 1 of 5 vowels (U, O, E, A, I). All stimuli were created using the MATLAB toolbox GERT v1.20 (Demeyer & Machilsen, 2011). In randomly alternating blocks participants were asked to report whether the local (local attention condition) or global letter (global attention condition) was a vowel or not by pressing the ‘f’ or ‘j’ key. The local and global letter were either congruent (both vowels or consonants) or incongruent (one vowel, one consonant). A total of 100 experimental trials were presented to participants in addition to 10 practice trials. In each trial, a fixation cross was presented for 100 ms followed by a letter shape for 300 ms. Participants received a 4 stime limit starting at stimulus onset to provide a response. Accuracy and response times were registered and stimulus presentation and response registration were controlled by PsychoPy (Peirce, 2007).

##### Coherent Motion Task (CM).

An array of 600 moving dots was presented at central fixation to participants (Fig. 2C). A proportion of the dots moved in the same direction (*global motion*), while the other dots moved in random directions. The direction of global motion was manipulated and had four levels: up, down, left and right. The proportion of dots that moved in the global motion direction was manipulated and consisted of eight levels ranging from 5 to 80%. Each dot had a diameter of 0.28 cm and moved at a speed of 7.1 cm/s. All dots were presented in an array with a diameter of 15 cm. The stimuli were presented for 500 ms and the participants had to make a forced choice between the four possible motion directions by pressing one of the four arrow keys. A total of 400 experimental trials were presented and 80 practice trials. Stimulus presentation was controlled and accuracy of responses was registered using custom software written in C# developed in Visual Studio.

#### Procedure

Testing took place in multiple one-hour sessions for different groups each consisting of approximately 15 participants. Each participant performed the computer tasks individually on a Dell Inspiron desktop computer in a slightly darkened computer room. The monitor had a width of 46 cm and a height of 26 cm. Participants viewing distance was on average 45 cm, but was not constrained by a chin rest. The tasks were administered in fully counterbalanced order.

#### Data analysis method

To summarize performance on the L-EFT only the first response on each trial was used. Performance on the L-EFT was summarized by the proportion of correct responses and the median response times (RTs) of the accurate responses for each participant. Performance on the CM task was summarized by calculating the mean accuracy of each participant (*CM accuracy*). For the CM task no response times were registered. Performance on the NSAT was summarized by calculating the median RTs of accurate trials and the mean accuracy in the local (*NSAT local*) and global attention conditions (*NSAT global*).

### Results

#### Outliers

None of the participants obtained an accuracy below chance level on the L-EFT (.33). One participant obtained an accuracy near chance level (.53) on the global attention condition of the NSAT and was therefore excluded from subsequent analyses. No outliers were detected for accuracy in the CM task.

#### Reliability

The split-half Spearman-Brown reliability index and descriptive statistics of each measure are reported in Table 1. The split-half reliability of the L-EFT was moderate (.56) to good (.74) and the split-half reliability of the CM accuracy and NSAT local and global accuracy and RTs was good (range: .71–.96).

**Table 1:** Reliability estimates and descriptive statistics of each variable of the three studies.

Study	Task	Variable		Sample size^a^	*R*	*M*	*SD*
1	L-EFT	Accuracy		255	.56	.81	0.09
		RT			.74	2.70	1.07
	CM	Accuracy		235	.87	.67	0.10
	NSAT	RT	local	124	.96	0.53	0.07
			global		.95	0.52	0.07
		Accuracy	local		.71	.93	0.10
			global		.75	.92	0.10
2	L-EFT	Accuracy		246	.62	.86	0.08
		RT			.88	2.40	0.47
	RAPM	Accuracy		255	.76	.61	0.20
	Corsi	Memory span		132	.73	4.1	0.66
	Flanker	IE		130	.42	0.18	0.08
	Switching	Switch cost		130	.64	−0.70	0.27
3	L-EFT	Accuracy		167	.76	.85	0.36
		RT			.80	1.92	1.05
	RAPM	Accuracy			.67	.62	0.24
	Corsi	Memory span			.48	4.52	0.63
	G-EFT	Accuracy			.84	.75	0.23

**Notes.**

a Sample sizes represent the number of participants that performed the individual task on which reliability estimates were based and do therefore not match the sample size of the participants that performed all tasks relevant to the studies.

L-EFT Leuven Embedded Figures Test RT response times of accurate trials in seconds *R* Spearman-Brown split-half reliability *M* mean *SD* standard deviation NSAT Navon Selective Attention Task RAPM Raven Advanced Progressive Matrices Corsi Corsi tapping test IE Flanker incongruency effect G-EFT group Embedded Figures Test

#### Main analysis

**Figure 3:**
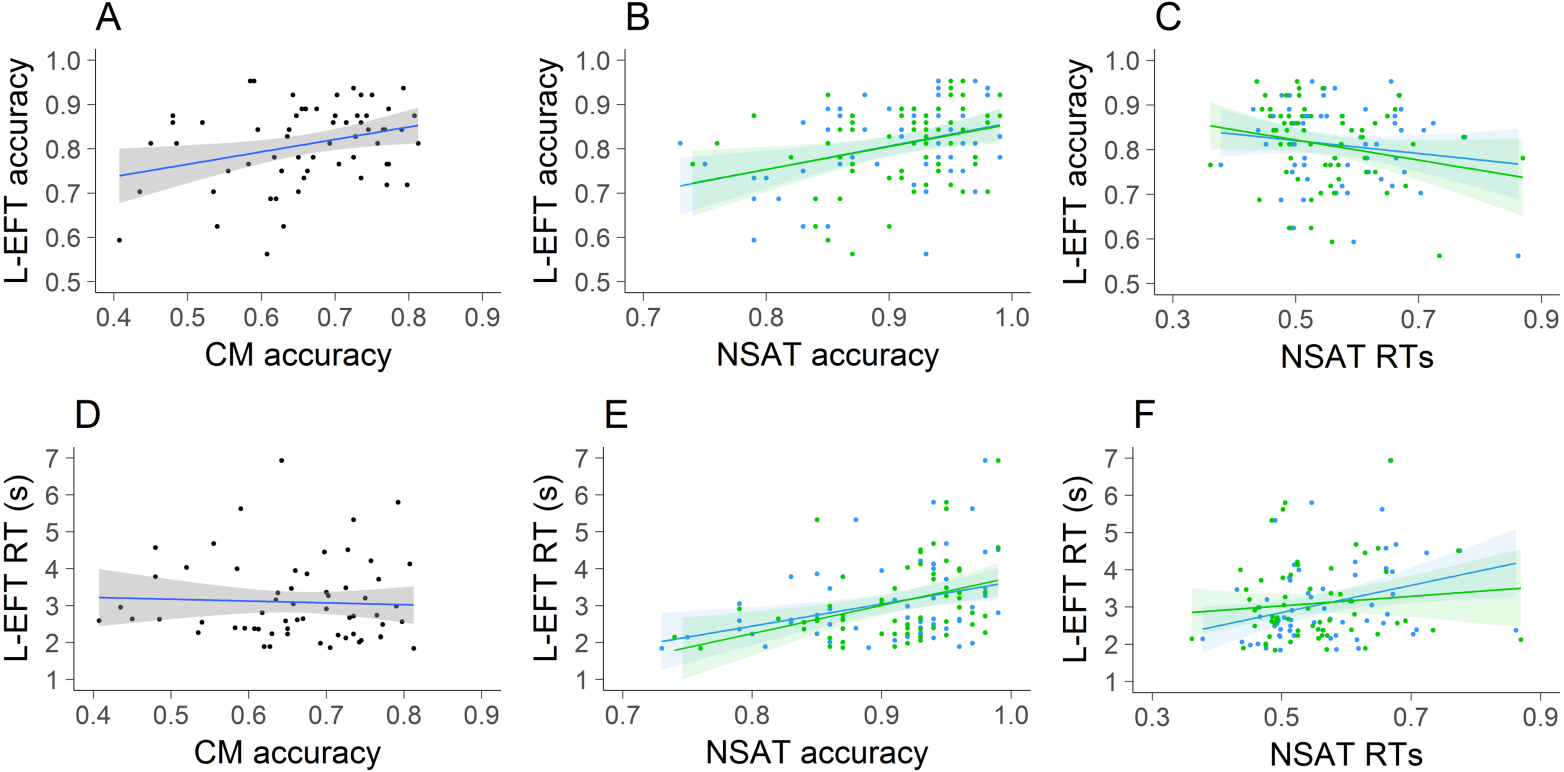
Visualization of results of Study 1. The scatterplots of L-EFT accuracy (A–C) and L-EFT RTs (D–F) with the CM accuracy (A, D), NSAT accuracy (B, E) and NSAT RTs (C, F). For the NSAT (B–F), the local condition is presented in the blue color and the global condition is presented in the green color.

Linear regression models were used to test to what extent L-EFT accuracy and RTs could be predicted by CM accuracy and NSAT local and global accuracy and RTs. All effects were evaluated against an alpha level of .05. All analyses were performed in R (R Core Team, 2016). The results of these models are reported in Table 2 and scatterplots between the outcome variable and each predictor are visualized in Fig. 3. The overall contribution of the predictors ranged between 18% to 24% of explained variance for the prediction of RTs and accuracy, respectively. For the prediction of the L-EFT accuracy only the NSAT local accuracy and NSAT global RTs reached significance at the level of .05. The results indicated that higher accuracy on the NSAT local condition and faster responses on the NSAT global condition were associated with higher L-EFT accuracy. For the prediction of the L-EFT RTs the only predictor that reached significance was the NSAT local RTs. Faster responses on the NSAT local condition were associated with faster responses on the L-EFT.

**Table 2:** Linear regression models of Study 1.

	L-EFT Accuracy			L-EFT RTs		
	Bèta	*T*	*P*	Bèta	*T*	*P*
Intercept	0.15	0.74	.46	−0.67	−0.85	.40
CM accuracy	0.19	1.72	.09	0.14	0.31	.76
NSAT global accuracy	0.21	0.71	.48	1.82	1.56	.12
NSAT local accuracy	0.62	2.44	**.02**	0.14	0.13	.89
NSAT global RT	−0.63	−2.19	**.03**	−1.29	−1.14	.26
NSAT local RT	0.18	0.68	.50	3.10	2.90	**<.01**
	Adjusted R-squared: .24			Adjusted R-squared: .18		

**Notes.**

Bolded values indicate the significant values at an alpha level of .05.

L-EFT RTs, response times of accurate trials in seconds Bèta linear regression coefficient *T* *t* value *P* *p* value

### Discussion

In Study 1, we aimed to evaluate to what extent perceptual ability could account for individual differences in performance on the L-EFT. For that reason, we selected a coherent motion task and a variant of the Navon hierarchical letter task to evaluate to what extent performance on the L-EFT could be predicted by performance on these two tasks. No evidence was found for a high amount of predictive power for the CM task and the NSAT. There was no clear pattern showing that performance in either the local or global attention condition of the NSAT was a better predictor of L-EFT performance. In addition, CM accuracy was not a significant predictor of L-EFT performance. Thus, these results further support the finding that there is little convergence between different measures that are all considered to measure local/global perceptual style in student samples (Chamberlain et al., 2017; Milne & Szczerbinski, 2009).

## Study 2

While Study 1 set out to improve our understanding of the relationship between different tasks that are assumed to measure local or global perceptual styles, it did not provide us with an answer to the question whether the L-EFT is related to fluid intelligence and/or EFs. Therefore, in Study 2 the relationship between L-EFT performance, three executive sub-domains and fluid intelligence was evaluated. The three EF domains were derived from the framework reviewed by Diamond (2013): inhibitory control, working memory and cognitive flexibility. Each of these cognitive abilities have the potential to play a role in EFT performance. Good performance on the EFT could be related to inhibitory control, as one may need to inhibit or suppress the surrounding (embedded) context (Brosnan et al., 2002). Cognitive flexibility could be important in the EFT, as switching between local or global stimulus attributes of the embedded figures could facilitate target detection. Short-term memory could be required to keep the target in mind whilst searching the embedding contexts. Finally, the general task demands (setting up goals and sub-goals, and executing each in the correct order) could depend on the same capacities measured in typical fluid intelligence tests (Duncan, 2013).

### Methods

#### Participants

A new sample of 132 undergraduate psychology students participated in this study for course credits. The median age was 18 years (*SD* = 1.44). The sample was primarily female (90%). A subset of 45 participants completed the L-EFT twice with a two-week interval in between the test and retest. These participants were also undergraduate psychology students with a median age of 19 years (*SD* = 3.74) and they were primarily female (96%). All procedures performed in this study were in accordance with the ethical standards of the institutional ethical committee and (approval number: S58409) and in accordance with the 1964 Helsinki declaration. Written informed consent for each participant was obtained prior to testing.

#### Instruments

##### Leuven embedded figures task.

The L-EFT as described in Study 1 and visualized in Fig. 2A was used in this study.

##### Raven Advanced Progressive Matrices short form (RAPM).

A computerized version of the short form of the original RAPM was administered to participants. The RAPM has good psychometric properties and allows for a quick assessment of fluid intelligence (Arthur & Day, 1994). Participants were presented with a 3 by 3 matrix of visual designs and asked to detect the rule that determined the matrix. They needed to select one response alternative out of 8 possible alternatives which would complete the matrix best. The matrix of visual designs had a size of 15 cm^2^ and the response options were presented just below the matrix and had a height of 10 cm and width of 20 cm. Participants received a 15-minute time limit to complete 12 test items, which were presented in order of progressive difficulty. Participants were not able to revisit previously answered items. Participants received a 5-minute warning, once the first 10 min of the test had passed. Both accuracy and response times were registered. Presentation of stimuli and response registration were controlled by PsychoPy (Peirce, 2007).

##### Corsi tapping test.

**Figure 4:**
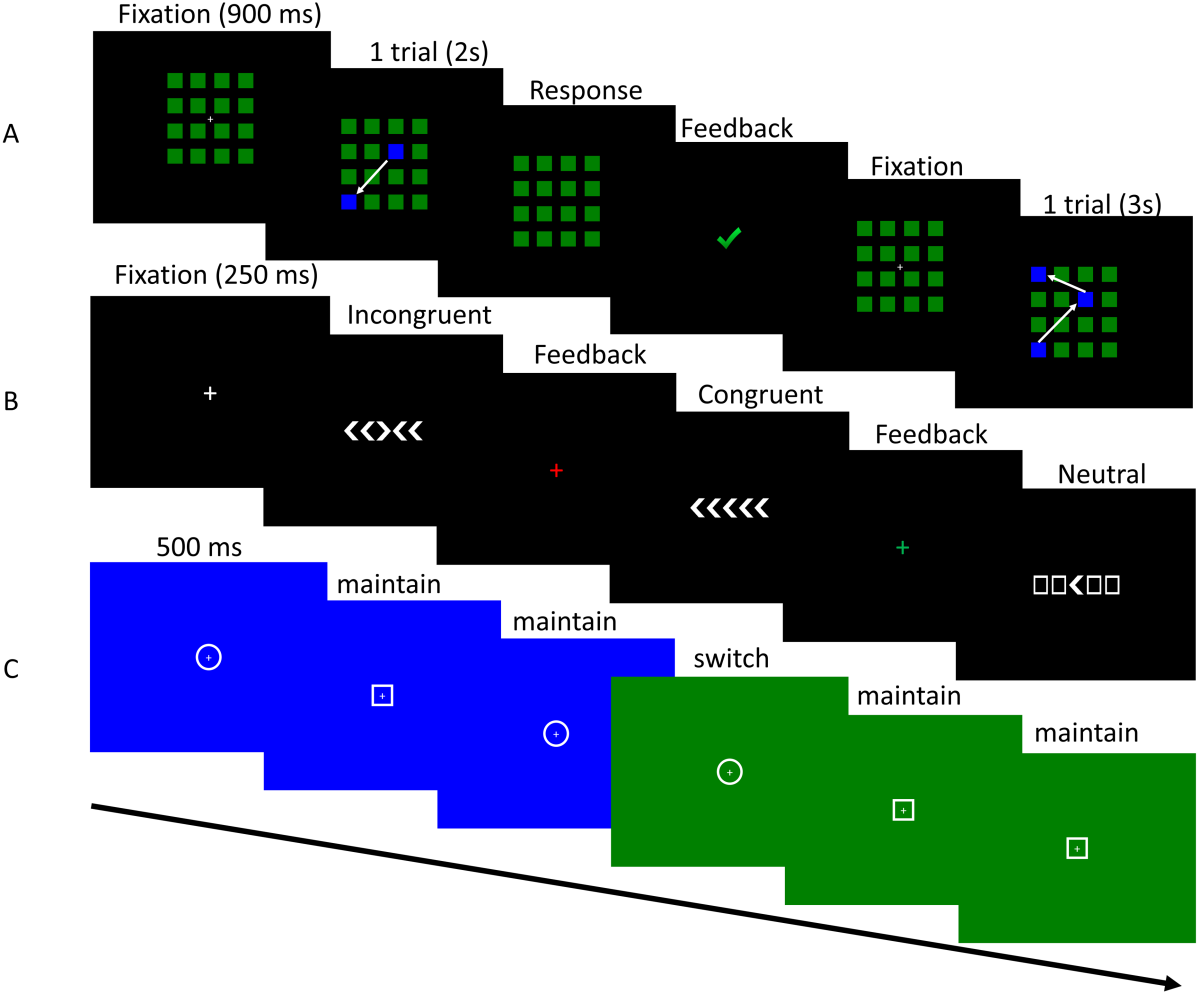
Visualization of the tasks used in Study 2. Task sequence of the Corsi tapping test (A), flanker task (B) and switching task (C).

A computerized version based on the original paradigm of the Corsi tapping test was administered to estimate short-term memory span (Corsi, 1973). A 4 by 4 matrix of green squares was presented in the centre of the screen on a black background. Each square had a size of 1.4 cm^2^ and the entire matrix of squares had a size of 6.65 cm^2^. A particular number of squares sequentially changed colour to blue (1s) and the order of these colour-changing squares was determined at random (Fig. 4A). Participants had to click on the squares in the same order as the presented sequence after the entire sequence was presented. During stimulus presentation, the cursor was disabled to prevent participants from tracking the stimulus presentation with their cursor to aid their memory. Participants could not proceed to the next trial without providing a response. The sequence length of each trial was determined by a 1-up 2-down staircase that started at a sequence length of 2 items. Five practice trials were presented. An additional 32 trials were administered to estimate the maximum sequence length an individual could reproduce. Presentation of stimuli and response registration were controlled by PsychoPy (Peirce, 2007).

##### Flanker task.

The flanker task used in this study was based on the paradigm originally developed by Eriksen & Eriksen (1974). A white arrow (target) was presented in the middle of the screen and two flankers (arrows or squares) were presented on both sides of the target for 250 ms. The arrows and squares had a height of 2.5 cm. Participants were asked to respond as quickly as possible with the arrow keys on the keyboard in which direction the white arrow was pointing. The task had to be performed in three conditions that were presented in a random order: congruent, incongruent and neutral flankers (Fig. 4B). In the congruent condition the flankers and target arrow were pointing in the same direction, while in the incongruent condition the flankers were pointing in the opposite direction as the target arrow. In the neutral condition the flanker stimuli were squares. A total of 120 experimental trials and 20 practice trials were performed by each participant. Between trials a fixation cross was presented for 250 ms and the colour of the fixation cross was changed to red or green to provide feedback to participants. After half of the trials a pause screen appeared and participants could decide when they continued with the second half of the trials. Reaction times and accuracy were registered. Stimulus presentation and response registration were controlled by PsychoPy (Peirce, 2007).

##### Switching task.

A cued task switching paradigm with an arbitrary stimulus–response association was used (Kiesel et al., 2010). A white circle or a white square with a diameter of 3.5 cm was presented in the middle of the screen (target) on a blue or green background for 500 ms (Fig. 4C). Participants were asked to respond as quickly as possible with the “up” key for the circle and with the “down” key for the square when the background colour of the screen was green and had to reverse this stimulus-response rule when the background colour of the screen was blue. The stimulus-response rule switched unpredictably after 6 to 12 trials. A total of 20 switches occurred during the task. Accuracy and reaction times for each trial were registered. Presentation of stimuli and response registration were controlled by PsychoPy (Peirce, 2007).

#### Procedure

Testing took place during multiple one-hour sessions for different groups each consisting of approximately 15 participants. All tasks were presented on a monitor with a width of 46 cm and a height of 26 cm in a slightly darkened computer room. The average viewing distance was approximately 45 cm. The RAPM was always administered as the first test. After the RAPM, in half of the sessions the L-EFT was administered as the second task and followed by a block of EF tasks, while in the other half of the sessions the block of EF tasks was administered before the L-EFT. The EF tasks were always administered in the same order: switching task, flanker task and the Corsi tapping test.

#### Data analysis method

Performance on the L-EFT was summarized by calculating the mean accuracy and median RTs on accurate trials. To summarize flanker performance, the RTs for accurate trials of the congruent and incongruent condition were divided by the RTs for accurate trials on the neutral condition. Then, a difference score was calculated based on the normalized data between the incongruent and congruent condition (*flanker incongruency effect)*. Performance on the RAPM was summarized using the proportion of correct items for each participant. Performance on the Corsi tapping test was summarized by calculating the average of the number of correctly reproduced items of each sequence (*memory span)*. Performance on the switching task was summarized by calculating the difference between the normalized RTs for accurate trials on switch (trials immediately after a cued task switch) versus maintain trials, which will be referred to as *switch cost*.

### Results

#### Outliers

No participant obtained an accuracy below chance level on the L-EFT (.33). For the flanker task, data of four participants that had an accuracy 2.5 standard deviations below the mean of the congruent and neutral conditions were removed. For the RAPM, data of two participants were excluded, because their performance was fast and inaccurate on the RAPM. They had an average reaction time of two standard deviations below the mean in combination with an accuracy one standard deviation below the mean. There were no outliers detected on the memory span index. For the switching task, three participants who obtained an accuracy level below or equal to chance level in the maintain trials were excluded from subsequent analyses. Additionally, one individual who obtained a mean accuracy on the maintain trials that lay 2.5 standard deviations below the mean was excluded from further analyses. Based on these criteria 8.5% of the data was excluded from further analyses.

#### Reliability

The reliability of all measures estimated with the Spearman-Brown split-half method and descriptive statistics of all measures are reported in Table 1. Overall, the reliability of the measures was moderate to good (range: .62–.88) except for the flanker incongruency effect, which had a lower split-half reliability (.42). Additionally, the test-retest reliability of the L-EFT was estimated by calculating the intra-class correlation on a sample of 45 participants who completed the L-EFT twice. The test-retest reliability of accuracy (*r*(44) = .55, 95% CI [.30–.72]) and RTs (*r*(44) = .45, 95% CI [.19–.66]) was moderate.

#### Main analysis

**Figure 5:**
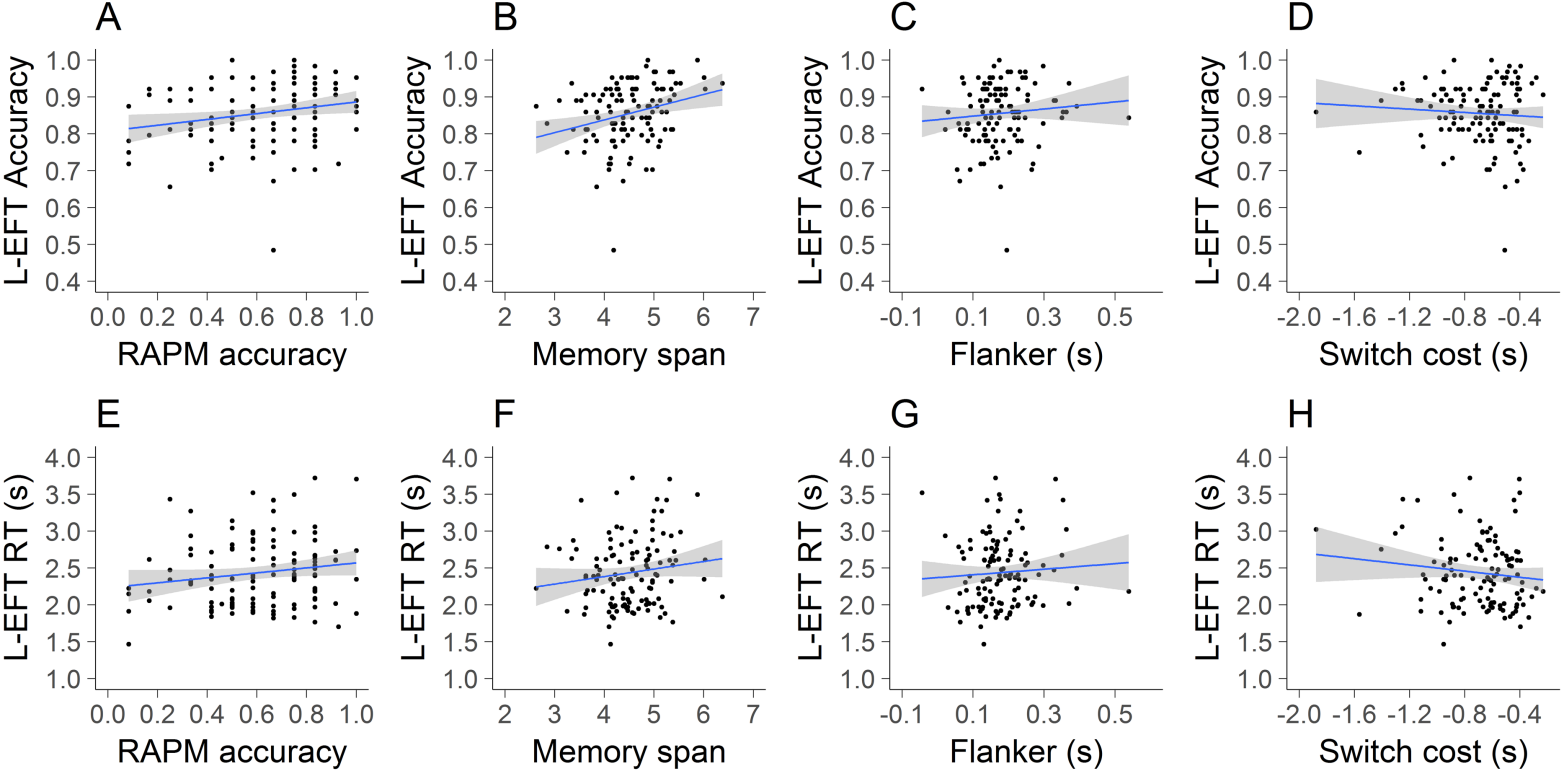
Visualization of results of Study 2. The scatterplots of L-EFT accuracy (A–D) and L-EFT RTs (E–H) with the RAPM accuracy (A, E), memory span (B, F), flanker incongruency effect (C, G) and switch cost (D, H). The blue line represents the linear trend and the grey area the standard error.

Linear regression models were used to predict L-EFT accuracy and RTs by performance on the flanker task, Corsi tapping test, RAPM and switching task (Table 3). Scatterplots of the outcome variables and each predictor are also presented in Fig. 5. The results indicated that the main effect of the RAPM, flanker incongruency effect and switch cost did not have a significant influence on L-EFT accuracy. Memory span contributed significantly at the .05 level for the prediction of L-EFT accuracy. For the prediction of L-EFT RTs there were no significant predictors. Overall, the explained variance was quite low, ranging between 2% for the prediction of RTs to 7% for the prediction of accuracy.

### Discussion

We studied the contribution of EFs and IQ to the L-EFT in a large sample of undergraduate psychology students. It was predicted that EFT performance could relate to intelligence, cognitive flexibility, short-term memory span and inhibitory control. In order to measure intelligence and EFs the following battery of tests was administered: RAPM short form, switching task, Corsi tapping test and the flanker task. Little support was found for a significant contribution of EFs and IQ to performance on the L-EFT, except for a small but significant association between memory span and L-EFT accuracy. These results stand somewhat in contrast to the studies that have linked the original EFT to EFs and fluid intelligence (Brosnan et al., 2002; McKenna, 1984).

**Table 3:** Linear regression models of Study 2.

	L-EFT Accuracy			L-EFT RT		
	Bèta	*T*	*P*	Bèta	*T*	*P*
Intercept	0.66	11.36	<.001	1.71	5.092	<.001
RAPM	0.05	1.28	.20	0.26	1.27	.21
Memory span	0.03	2.49	**.02**	0.08	1.19	.24
Flanker IE	0.03	0.35	.73	0.06	0.11	.92
Switch cost	−0.04	-1.3	.20	−0.26	-1.61	.11
	Adjusted R-squared: .07			Adjusted R-squared: .02		

**Notes.**

Bolded values indicate the significant values at an alpha level of .05.

Flanker IE Flanker Incongruency Effect L-EFT RT response times of accurate trials in seconds Bèta linear regression coefficient *T* *t* value *P* *p* value

Brosnan et al. (2002) found that dyslexic children and adults with poor working memory ability performed worse on the G-EFT and interpreted this finding as a reflection of poor ability to disinhibit the surrounding task-irrelevant context while searching for the target shape. Although it could be that in samples with EF dysfunctions EF skills may affect EFT performance, our results do not support the idea that good performance on an embedded figures task is necessarily a good proxy of inhibitory skills. Therefore, it seems premature to interpret performance on the EFT as a reflection of poor inhibitory skills since there is little empirical support for this idea.

McKenna (1984) summarizes the results of a number of studies that reported correlations between the performance on various forms of the original EFT and the Raven progressive matrices. These studies involved cross-cultural, children, elderly and student samples. The correlations between the RAPM and EFT ranged from .40 to .78 (*M* = .56, *SD* = 0.11) and were estimated on sample sizes ranging from 30 to 173 participants (*M* = 63, *SD* = 32.82). Given the robust findings of a moderate to good correlation between fluid intelligence and EFT performance across many different samples and the fact that accuracy spanned the entire range of the RAPM in our sample, our results showing little correlation between the RAPM and L-EFT contrast the earlier findings reported in McKenna (1984).

A possible explanation for the difference in findings on the contribution of fluid intelligence to EFT performance between our study and the studies reported in McKenna (1984) is that the new L-EFT may be more independent of fluid intelligence than the original EFT developed by Witkin. Because the perceptual factors that make the target shape more embedded in the L-EFT are systematically manipulated, the performance on the L-EFT might be driven to a larger extent by pure perceptual factors than by general problem solving skills. In Study 3 we tested this possible explanation by directly comparing the contribution of fluid intelligence to the performance on the L-EFT and G-EFT. In addition, we compared the contribution of memory span to performance on the L-EFT and G-EFT since we predicted that the L-EFT would be less affected by participant’s memory span than the G-EFT.

## Study 3

To test whether the L-EFT has a smaller association with memory span and fluid intelligence than the G-EFT, we directly compared the contribution of these two predictors to performance on the G-EFT and L-EFT in Study 3.

### Methods

#### Participants

A new sample of 167 psychology students participated in this study for course credits. Their median age was 18 years (*SD* = 1.05). The sample was primarily female (82%). A large subset of these participants (*n* = 107) completed the L-EFT twice at two different time points (median age=18 years, *SD* = 0.93 years, 78% females). All procedures performed in this study were in accordance with the ethical standards of the institutional ethical committee as in Study 1 and 2. Written informed consent for each participant was obtained prior to testing.

#### Instruments

##### The leuven embedded figures test.

The task procedure of the L-EFT was slightly adjusted to increase the reliability of the task. The target, embedding context and distractors were also presented simultaneously as in Study 1, but for a limited duration of 3 s. After the stimulus presentation, subjects had to make a forced choice between the 3 response alternatives by pressing a button on the numeric keypad. Unlike the task procedure of the L-EFT in the previous studies, subjects were not allowed to provide multiple responses for each trial and no feedback was provided. All 64 figures of the stimulus set of the L-EFT were presented twice (128 trials). The stimulus presentation and response registration were controlled using custom software developed with PsychoPy (Peirce, 2007).

##### The Group Embedded Figures Test (G-EFT, Witkin et al., 1971).

The G-EFT was administered to participants in group. Participants were asked to trace the outline of a target shape hidden within a complex figure. On the first pages of the test booklet participants received instructions on how to complete the task. They were instructed that the test aims to measure their ability to find a simple form when it is hidden in a complex pattern. They could see an example of a target shape and its location in a complex shape and they were instructed that the simple form would always be present in the complex shape with the same proportions, size and direction as the original simple form. They were instructed not to skip items and were allowed to correct mistakes. As in the original version, participants were instructed to complete seven practice trials within 2 min and two sets of 9 test trials each within 5 min. On each page of the test booklet two context shapes are presented. Underneath each context shape a text indicated which of the 8 possible target shapes the participant had to trace in the context shape. The target shapes were located on the last page of the booklet. The accuracy of the responses was recorded.

##### Executive functions.

Fluid intelligence was estimated with the RAPM administered in the same way as described in Study 2. Short-term memory span was estimated with the Corsi tapping test. The number of trials of the Corsi tapping test was reduced from 32 (in Study 2) to 20 to avoid that fatigue would build up during the task.

#### Procedure

Testing took place during multiple one-hour sessions for different groups each consisting of approximately 15 participants. Each participant performed the computer tasks individually on a Dell Inspiron desktop computer in a slightly darkened computer room. The monitor had a width of 46 cm and a height of 26 cm. The viewing distance was approximately 45 cm. All four tasks were administered in fully counterbalanced order. The G-EFT was administered exactly according to the instructions in the manual (Witkin et al., 1971).

### Results

#### Outliers

For the RAPM and Corsi tapping test no outliers were detected according to the criteria used in Study 2. For the G-EFT, no outliers were detected. To evaluate the test-retest reliability, one outlier was removed from the data who performed below chance level (<.33) on the L-EFT in the first session of data collection. None of the participants obtained an accuracy below chance level (.33) on the L-EFT that was administered in the second session of data collection.

#### Reliability

The reliability of all measures estimated with the split-half Spearman-Brown method ranged from moderate to good (range: .48–.84, Table 1). In addition, descriptive statistics of each measure are reported in Table 1. The split-half reliability of the L-EFT was good (range: .76–.80). The test-retest reliability was estimated by calculating the intra-class correlation of accuracy on the L-EFT of the participants who completed the L-EFT in two test sessions that were 3 months apart and this indicated a moderate test-retest reliability for accuracy, *r*(105) = .46, 95% CI [.30–.60] and a weak test-retest reliability for median RTs, *r*(105) = .21, 95% CI [.02–.38].

#### Main analysis

**Figure 6:**
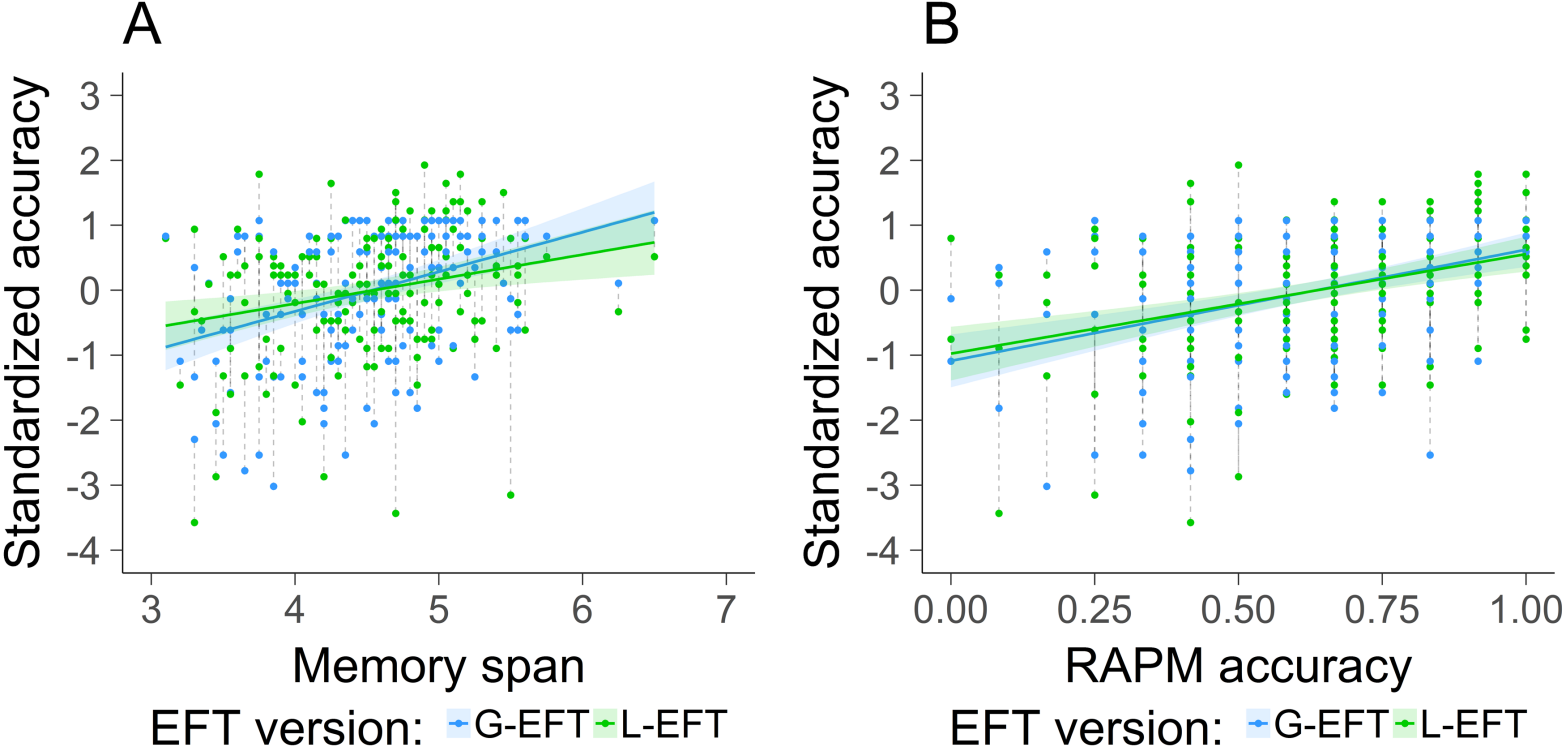
Visualization of the results of Study 3. The standardized accuracy of the G-EFT (blue) and L-EFT (green) as a function of memory span (A) and accuracy on the RAPM (B). The lines represent the linear trend and the areas around the line the standard error. The dashed lines connect the two observations of each subject.

A linear mixed model was used in which accuracy was predicted by the main effect of the type of EFT task, the main effect of the covariates RAPM and memory span, the interaction between the type of EFT task and RAPM and the interaction between the type of EFT task and the memory span, including a random intercept for the subjects. Since the L-EFT is associated with a guess rate of 33% and the G-EFT is not, the accuracy on both tasks is measured on a different scale. Therefore, to compare these two measures we standardized the accuracy. The model was fitted with the lme4 package of R (Bates et al., 2015, p. 4). The results are reported in Table 4 and visualized in Fig. 6. The main effect of the type of EFT task was not significant. The main effects of RAPM and memory span were significant, indicating that good performance on the EFT, independent of the type of EFT task, is positively associated with good performance on the RAPM and memory span. Furthermore, the pairwise interactions between the EFT version and RAPM and memory span were not significant. The latter results indicate that the RAPM and memory span had a similar impact on the standardized accuracy for the L-EFT compared to the G-EFT.

**Table 4:** Linear mixed model of Study 3.

Predictor	Bèta	*T*	*P*
Intercept	−3.18	−6.15	<.001
EFT version	1.06	1.79	.07
Memory span	0.50	−1.74	<.001
RAPM	1.44	4.75	<.001
EFT version * Memory span	−0.23	−1.74	.08
EFT version * RAPM	−0.06	-0.16	.87

**Notes.**

EFT version, L-EFT is coded as 1 and group EFT (G-EFT) as 0.

### Discussion

The results revealed that the standardized performance on the G-EFT and L-EFT was similarly associated with memory span and fluid intelligence. We had predicted that memory span would affect the performance on the G-EFT more than on the L-EFT because the target shape is presented simultaneously with the embedding contexts in the L-EFT and not in the G-EFT. In addition to that, we tested whether fluid intelligence was more strongly related to performance on the G-EFT than the L-EFT since we found smaller associations between fluid intelligence and L-EFT performance in Study 2 than what is typically reported in the literature about the G-EFT (McKenna, 1984). However, we did not find support for a stronger association between performance on the RAPM and the G-EFT than the RAPM and the L-EFT. This result indicates that good performance on the G-EFT does not require more general problem solving skills than the L-EFT.

## Conclusion

Embedded figures tests have been interpreted as a measure of disembedding or local perceptual style, as well as a measure of fluid intelligence, inhibitory control and cognitive flexibility (Brosnan et al., 2002; Duncan, 2013; Goodenough & Karp, 1961; Roberge & Flexer, 1981). In order to evaluate to what extent performance on the embedded figures test can be explained by these different functions, we tested to what extent a new embedded figures test, the L-EFT, was associated with different measures of local/global perceptual style, executive functions and fluid intelligence. We also compared the L-EFT to the most commonly used traditional version of the EFT (Witkin et al., 1971). It has been shown that perceptual grouping affects embeddedness of shapes (De Wit et al., 2017) and that variants of this task correlated poorly with ostensive measures of local and global visual processing (Chamberlain et al., 2017). However, these studies did not reveal the relation between the EFT, fluid intelligence and executive functions. In the current studies, we tested how individual differences in performance on the L-EFT and G-EFT relate to performance on other tasks that are often assumed to measure local/global perceptual styles and measures of fluid intelligence and executive functions.

In Study 1, we demonstrated that inter-individual differences on the embedded figures task, Navon selective attention and coherent motion task are not strongly associated to each other. This finding aligns with the results of the study of Milne & Szczerbinski (2009) and Chamberlain et al. (2017), which indicated that there is no single common factor underlying performance in tasks interpreted as measuring local/global perceptual style.

In addition to interpreting the EFT as a measure of local/global perceptual style, better performance on the EFT has also been interpreted as a reflection of better executive functions (Brosnan et al., 2002). However, to our knowledge, no studies had explicitly tested whether executive functions can predict EFT performance. Thus, these interpretations were based on intuitions about the cognitive functions required to complete the test items. Therefore, in Study 2 and 3 we explicitly tested to what extent executive functions and fluid intelligence predict performance on the L-EFT and in Study 3 we compared the contribution of memory span and fluid intelligence to performance on the L-EFT and the G-EFT. Study 2 revealed that L-EFT performance was quite independent of fluid intelligence, memory span, inhibition and cognitive flexibility. Study 3 revealed that fluid intelligence and memory span are significantly associated with good EFT performance and that this association was not moderated by the EFT version.

We also evaluated the reliability of the L-EFT. The split-half reliability of the L-EFT indicates that people perform relatively consistently on the two test halves. However, the stability of the scores over a period of two weeks in Study 2 and 4 months in Study 3 was only moderate. This raises the question whether the L-EFT measures a stable trait within a sample of undergraduates. It has been shown that performance on a battery of tests to measure field- dependence/independence remained stable throughout development (Witkin, Goodenough & Karp, 1967) and a good test-retest correlation (ranging from .78 to .92) at test-retest intervals ranging from 1 h to 6 weeks has been reported for the G-EFT in young, primarily female student samples (Kepner & Neimark, 1984). In addition, for other perceptual tasks such as the Navon task researchers have reported high test-retest reliability over a period of 7 to 10 days when using a hierarchical shape task but lower test-retest reliability in a traditional Navon letter task. These results suggest that perceptual biases can be stable across time, but that this stability is impacted by differing task demands which can create noise in the participant’s response (Dale & Arnell, 2013). The fact that stability may differ according to task demands should motivate caution in researchers studying individual differences in perceptual ability. Whilst the reliability of personality traits has received much attention (e.g., Roberts & DelVecchio, 2000), less attention has been paid to the reliability of perceptual traits, and measures of stability should be included in future investigations.

We must consider one possible limitation to the generalizability of our results. We tested a primarily female student sample in our three different studies that may be more homogeneous regarding their perceptual abilities, fluid intelligence and executive functions compared to a random sample from the general population. Even though we attempted to compensate for the sample homogeneity by choosing tasks that would maximize inter-individual differences within this group the convergence between the different measures may still differ in different samples. The moderate stability of the L-EFT could also be related to the homogeneous sample that took part in our study and a more heterogeneous sample may elicit different levels of perceptual trait stability.

In conclusion, the data from Study 1 suggests that good performance on the embedded figures test cannot be used as a proxy for a general perceptual style. Study 2 suggests good embedded figures test performance is not associated with individual differences in inhibition or cognitive flexibility. Study 2 and 3 finds some association between memory span and embedded figures performance, and Study 3 finds an association between embedded figures performance and fluid intelligence. Thus, across these studies there are some significant correlates of embedded figures performance, but none of these account for a large amount of variance in performance. This indicates that performance on the embedded figures tests cannot be assumed to be a good proxy for a general perceptual style, fluid intelligence, memory span, inhibition or cognitive flexibility. This means that deficits (or improvements) in embedded figures test performance in different clinical groups need to be interpreted with care, because we cannot definitively link performance with embedded figures to general perceptual or cognitive mechanisms.

##  Supplemental Information

10.7717/peerj.4524/supp-1 Supplemental Information 1 Script to calculate reliability of L-EFT

10.7717/peerj.4524/supp-2 Supplemental Information 2 Script to analyze data of study 1

10.7717/peerj.4524/supp-3 Supplemental Information 3 Script to analyze reliability of measures of switching task used in study 2

10.7717/peerj.4524/supp-4 Supplemental Information 4 Script to summarize data

10.7717/peerj.4524/supp-5 Supplemental Information 5 Script to analyze data of study 2

10.7717/peerj.4524/supp-6 Supplemental Information 6 Script to calculate reliability of the flanker task used in study 2

10.7717/peerj.4524/supp-7 Supplemental Information 7 Script to analyze data of study 3

10.7717/peerj.4524/supp-8 Supplemental Information 8 Script to analyze reliability of measures in study 1

10.7717/peerj.4524/supp-9 Supplemental Information 9 Raw data of study 1

10.7717/peerj.4524/supp-10 Supplemental Information 10 Raw data of study 2

10.7717/peerj.4524/supp-11 Supplemental Information 11 Data of study 3

## Additional Information and Declarations

### Data Availability

The following information was supplied regarding data availability: The raw data files and the scripts to analyze the data are provided as Supplemental Files.
