# Supplementary material for: The Leuven Embedded Figures Test (L-EFT): measuring perception, intelligence or executive function?
Source: PeerJ. 2018 Mar 26;6:e4524. doi: 10.7717/peerj.4524 (PMC5875396; doi:10.7717/peerj.4524)
Supplement: Supplemental Information 9 [file peerj-06-4524-s009.zip › Study 1/EFT/L-EFT_Practice/Rplot_pracTrialsCorrect_histogram.pdf]

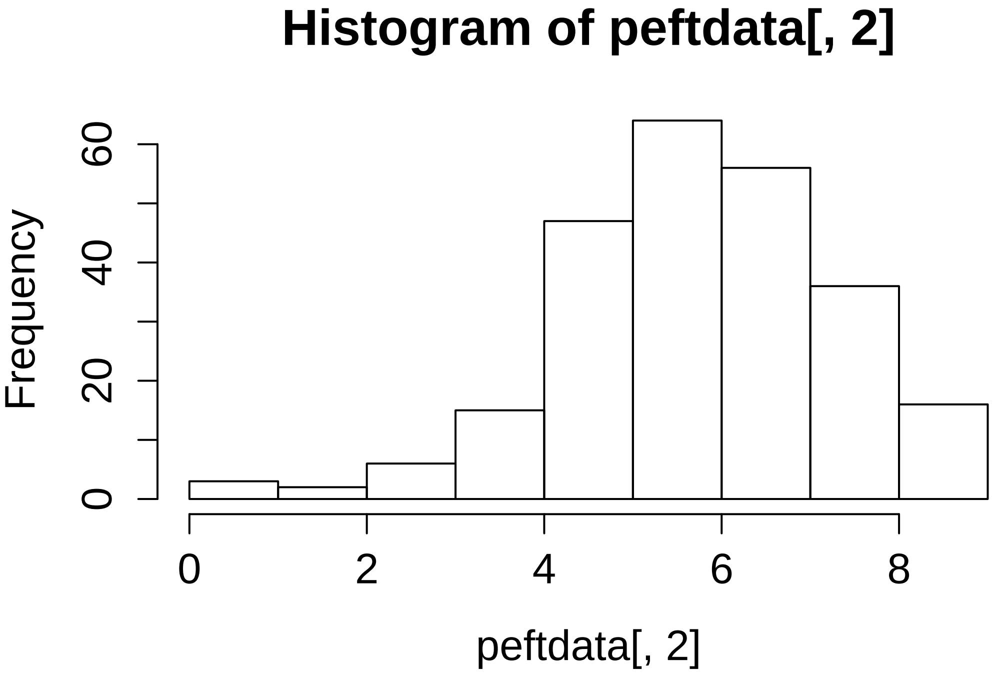
Histogram of peftdata[, 2]
Frequency
0
20
40
60
0
2
4
6
8
peftdata[, 2]
